# Late-onset isolated adrenocorticotropic hormone deficiency caused by nivolumab: a case report

**DOI:** 10.1186/s12902-019-0335-x

**Published:** 2019-02-19

**Authors:** Ayumu Takeno, Masahiro Yamamoto, Miwa Morita, Sayuri Tanaka, Ippei Kanazawa, Mika Yamauchi, Sakae Kaneko, Toshitsugu Sugimoto

**Affiliations:** 1 Internal Medicine 1, Shimane University Faculty of Medicine, 89-1, Enya-cho, Izumo, Shimane 693-8501 Japan; 2 Department of Dermatology, Shimane University Faculty of Medicine, 89-1, Enya-cho, Izumo, Shimane 693-8501 Japan

**Keywords:** Anti-programmed cell death protein 1 antibody, Anti-cytotoxic T-lymphocyte-associated protein 4 antibody, Nivolumab, Isolated adrenal deficiency, Thyroid dysfunctions

## Abstract

**Background:**

Immune checkpoint inhibitors including nivolumab, an anti-programmed cell death protein 1 antibody, are recently developed cancer immunotherapy agents. Immune checkpoint inhibitors are known to cause autoimmune-related side effects including endocrine dysfunctions. However, there are few reports on late-onset isolated adrenocorticotropic hormone (ACTH) deficiency caused by nivolumab.

**Case presentation:**

The patient was a 72-year-old female. When she was 64 years old, she was diagnosed with malignant melanoma of the left thigh accompanied by left inguinal lymph node metastases, and she received several courses of chemotherapy for malignant melanoma followed by the resection of these lesions. At 71 years of age, multiple metastases were found and treatment with nivolumab 2 mg/kg every 3 weeks was initiated. Six months later, replacement with levothyroxine was started because of hypothyroidism following mild transient thyrotoxicosis. Eleven months after the beginning of nivolumab, the treatment was discontinued because of tumor expansion. Four months after the discontinuation of nivolumab, general malaise and appetite loss worsened, and 2 months later, hyponatremia (Na; 120–127 mEq/L) and hypoglycemia (fasting plasma glucose; 62 mg/dL) appeared. Her ACTH and cortisol levels were extremely low (ACTH; 9.6 pg/mL, cortisol; undetectable). Challenge tests for anterior pituitary hormones showed that responses of ACTH and cortisol secretion to corticotropin-releasing hormone were disappeared, although responses of other anterior pituitary hormones were preserved. Thus, she was diagnosed with isolated ACTH deficiency. Her symptoms were improved after treatment with hydrocortisone.

**Conclusions:**

The present report showed a case of late-onset isolated ACTH deficiency accompanied by hyponatremia, which was diagnosed 6 months after the discontinuation of nivolumab. The effects of nivolumab last for a long time and the side effects of nivolumab can also appear several months after discontinuation of the drug. Repeated monitoring of serum sodium levels may be a beneficial strategy to find the unexpected development of adrenal insufficiency even after discontinuation of nivolumab.

## Background

Immune checkpoint inhibitors including ipilimumab and tremelimumab, anti-cytotoxic T-lymphocyte-associated protein 4 (CTLA-4) antibodies, and nivolumab and pembrolizumab, anti-programmed cell death protein 1 (PD-1) antibodies, are recently developed cancer immunotherapy agents which activate T lymphocytes and enhance immune responses to cancers [1–4]. In addition, these drugs also cause immune responses to some specific organs and cause immune-related adverse events such as colitis, rash, and hepatitis [5, 6]. Immune checkpoint inhibitors also cause endocrine dysfunctions such as thyroid dysfunctions [2, 7–9], hypophysitis [2, 9, 10], and type 1 diabetes [11–14] during its treatment period.

We report a case of isolated adrenocorticotropic hormone (ACTH) deficiency, which was diagnosed 6 months after the discontinuation of nivolumab, in a patient with malignant melanoma.

## Case presentation

The patient was a 72-year-old female. When she was 64 years old, a poorly-marginated black legion was found in her left thigh, which was gradually enlarged. Three years after the appearance of the skin legion, skin biopsy was performed in our hospital and she was diagnosed with malignant melanoma. Positron emission computed tomography showed left inguinal lymph node metastases. She was treated with DAVFeron therapy (dacarbazine; 120 mg/m^2^/day at day 1–5, nimustine; 60 mg/m^2^/day at day 1, vincristine; 0.6 mg/m^2^/day at day 1, and interferon β; 3 million units/day at day 1–5), which was followed by resection of the skin legion and intra-pelvic lymph node dissection. At 71 years of age, liver metastases and intra-pelvic lymph node metastases appeared, thus treatment with nivolumab 2 mg/kg every 3 weeks was initiated (day X).

**Fig. 1:**
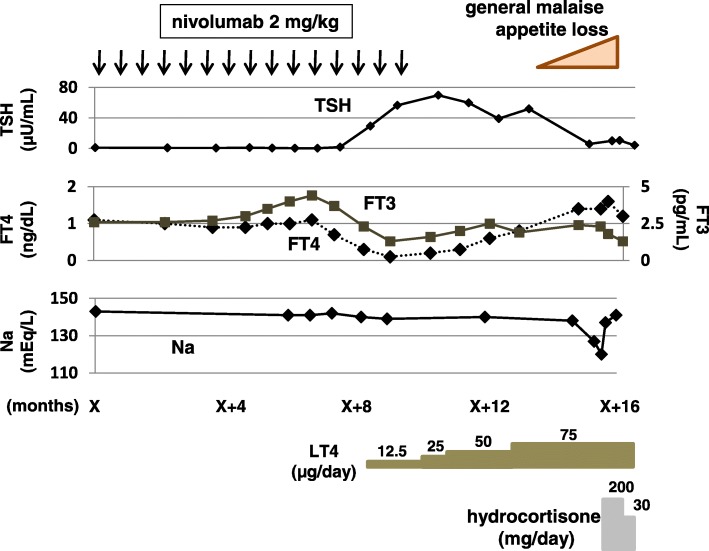
Clinical course and changes in the thyroid hormones, serum sodium levels

Six months after the day X, biochemical examination of blood revealed mild thyrotoxicosis, which did not need any medical treatment (Fig. 1). After that, hypothyroidism accompanied by general malaise appeared [thyroid-stimulating hormone (TSH); 29.3 μU/mL, free T3 (FT3); 2.3 pg/mL, and free T4 (FT4); 0.3 ng/dL] (Fig. 1). Anti-thyroperoxidase antibody and anti-thyroglobulin antibody were negative. She was diagnosed with primary hypothyroidism associated with nivolumab. Replacement with levothyroxine (LT4) was started, the dose was gradually increased to 75 μg/day, and thereafter her hypothyroidism was well-controlled (Fig. 1).

**Fig. 2:**
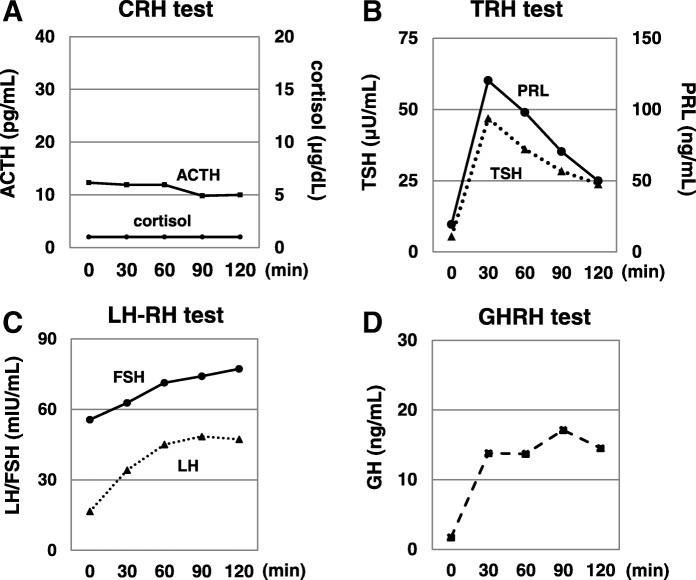
Results of challenge tests for anterior pituitary hormones. PRL: prolactin; LH: luteinizing hormone; FSH: follicle-stimulating hormone; GH: growth hormone; CRH: corticotropin-releasing hormone; TRH: thyrotropin-releasing hormone; LH-RH: luteinizing hormone-releasing hormone; GHRH: growth hormone-releasing hormone

Eleven months after the day X, the treatment was discontinued because of expansion of liver metastases. After that, best supportive care was performed to her disease. Four months after the discontinuation of nivolumab, general malaise and appetite loss appeared. Two months later, she was admitted to our hospital because these symptoms were worsened, which were accompanied by hyponatremia (Na 120–127 mEq/L) and hypoglycemia (fasting plasma glucose 62 mg/dL). Her ACTH and cortisol levels were low (9.6 pg/mL and undetectable, respectively). Challenge tests were performed to examine the secretion of anterior pituitary hormones. The responses of ACTH and cortisol to corticotropin-releasing hormone were disappeared, although the responses of other anterior pituitary hormones were preserved (Fig. 2). Thereby, she was diagnosed with isolated ACTH deficiency. Any lesion to cause hypopituitarism was not observed in the brain including the hypothalamus and the pituitary gland by enhanced computed tomography and magnetic resonance imaging. Her symptoms, hyponatremia, and hypoglycemia were rapidly improved after replacement of hydrocortisone (Fig. 1).

## Discussion and conclusions

Anti-CTLA-4 antibodies and anti-PD-1 antibodies are immunotherapy agents to interfere with tumor cell growth and survival by activating immune responses of T cells to cancer cells by blockade of signals via CTLA-4 and PD-1, respectively [15]. However, these drugs can also cause autoimmune disorders such as endocrine dysfunctions including hypopituitarism [2, 9, 10] and thyroid dysfunctions [2, 7–9]. The frequencies of hypophysitis were 0.3% and 11–15% in patients treated with nivolumab (anti-PD-1 antibody) [2] and ipilimumab (anti-CTLA-4 antibody) [16–18], respectively. In addition, several case reports reported a relationship between treatment with nivolumab and isolated ACTH deficiency [19–24]. These findings suggests that administration of nivolumab was associated with the thyroid dysfunctions and the isolated ACTH deficiency in our patient.

A notable finding in this case was that isolated ACTH deficiency appeared 6 months after the discontinuation of nivolumab. One clinical study showed that tumor progression of malignant melanoma was prevented for at least 16 weeks after discontinuation of nivolumab in 12 out of 17 patients [25]. Furthermore, Kimura et al. reported that two times administration of nivolumab for non-small cell lung cancer progressively shrank the primary legion of the tumor for 6 months [26]. These reports indicate that the anti-tumor effects of nivolumab last even after the discontinuation of the drug. In addition, Teramoto et al. reported that fulminant type 1 diabetes mellitus occured 6 weeks after the discontinuation of nivolumab [27]. This observation suggests that immune-related side effects can also develop after discontinuation of immune checkpoint inhibitors and that careful observation for endocrine disorders is recommended even after discontinuation of these agents.

Monitoring of serum sodium levels might help to detect unexpected adrenal insufficiency in patients treated with nivolumab. General malaise and appetite loss is considered as signs of adrenal insufficiency. Indeed, these symptoms appeared 2 months before the development of hyponatremia in this case, suggesting that careful observation or medical check for latent adrenal insufficiency need to be started when those signs are observed. Hyponatremia is one of the important laboratory findings of overt adrenal deficiency. Cho et al. reported that serum sodium levels were clearly decreased in three of the four patients with nivolumab-associated isolated ACTH deficiency [23], indicating that measurement of serum sodium levels may be useful to find adrenal insufficiency in patients treated with immune checkpoint inhibitors in clinical practice. In addition, hypopituitarism occurred 12–36 weeks after the initiation of nivolumab [19–24] and endocrine impairment potentially develops after discontinuation of the drug. These findings suggest that repeated monitoring of serum sodium levels, at least in the situation after the appearance of general malaise and appetite loss, may be effective to find the development of adrenal insufficiency in patients treated with nivolumab.

In conclusion, the present report showed a case of nivolumab-induced late-onset isolated ACTH deficiency accompanied by hyponatremia. The effects of immune checkpoint inhibitors last for a long time. This case teaches us that repeated monitoring of serum sodium levels may be a beneficial strategy to find the unexpected development of adrenal insufficiency in patients treated with immune checkpoint inhibitors even after these agents are discontinued.

## Abbreviations

ACTH: adrenocorticotropic hormone
CRH: corticotropin-releasing hormone
CTLA-4: cytotoxic T-lymphocyte-associated protein 4
FSH: follicle-stimulating hormone
FT3: free T3
FT4: free T4
GH: growth hormone
GHRH: growth hormone-releasing hormone
LH: luteinizing hormone
LH-RH: luteinizing hormone-releasing hormone
LT4: levothyroxine
PD-1: programmed cell death protein 1
PRL: prolactin
TRH: thyrotropin-releasing hormone
TSH: thyroid-stimulating hormone
